# Point-of-care screening for heart failure with reduced ejection fraction using artificial intelligence during ECG-enabled stethoscope examination in London, UK: a prospective, observational, multicentre study

**DOI:** 10.1016/S2589-7500(21)00256-9

**Published:** 2022-01-05

**Authors:** Patrik Bachtiger, Camille F Petri, Francesca E Scott, Se Ri Park, Mihir A Kelshiker, Harpreet K Sahemey, Bianca Dumea, Regine Alquero, Pritpal S Padam, Isobel R Hatrick, Alfa Ali, Maria Ribeiro, Wing-See Cheung, Nina Bual, Bushra Rana, Matthew Shun-Shin, Daniel B Kramer, Alex Fragoyannis, Daniel Keene, Carla M Plymen, Nicholas S Peters

**Affiliations:** a National Heart and Lung Institute and Centre for Cardiac Engineering, Imperial College London, London, UK; b Imperial College Healthcare NHS Trust, London, UK; c UKRI Centre for Doctoral Training in AI for Healthcare, Imperial College London, London, UK; d NHS Ealing Clinical Commissioning Group, London, UK; e Richard A and Susan F Smith Center for Outcomes Research in Cardiology, Beth Israel Deaconess Medical Center, Harvard Medical School, Boston, MA, USA

## Abstract

**Background:**

Most patients who have heart failure with a reduced ejection fraction, when left ventricular ejection fraction (LVEF) is 40% or lower, are diagnosed in hospital. This is despite previous presentations to primary care with symptoms. We aimed to test an artificial intelligence (AI) algorithm applied to a single-lead ECG, recorded during ECG-enabled stethoscope examination, to validate a potential point-of-care screening tool for LVEF of 40% or lower.

**Methods:**

We conducted an observational, prospective, multicentre study of a convolutional neural network (known as AI-ECG) that was previously validated for the detection of reduced LVEF using 12-lead ECG as input. We used AI-ECG retrained to interpret single-lead ECG input alone. Patients (aged ≥18 years) attending for transthoracic echocardiogram in London (UK) were recruited. All participants had 15 s of supine, single-lead ECG recorded at the four standard anatomical positions for cardiac auscultation, plus one handheld position, using an ECG-enabled stethoscope. Transthoracic echocardiogram-derived percentage LVEF was used as ground truth. The primary outcome was performance of AI-ECG at classifying reduced LVEF (LVEF ≤40%), measured using metrics including the area under the receiver operating characteristic curve (AUROC), sensitivity, and specificity, with two-sided 95% CIs. The primary outcome was reported for each position individually and with an optimal combination of AI-ECG outputs (interval range 0–1) from two positions using a rule-based approach and several classification models. This study is registered with ClinicalTrials.gov, NCT04601415.

**Findings:**

Between Feb 6 and May 27, 2021, we recruited 1050 patients (mean age 62 years [SD 17·4], 535 [51%] male, 432 [41%] non-White). 945 (90%) had an ejection fraction of at least 40%, and 105 (10%) had an ejection fraction of 40% or lower. Across all positions, ECGs were most frequently of adequate quality for AI-ECG interpretation at the pulmonary position (979 [93·3%] of 1050). Quality was lowest for the aortic position (846 [80·6%]). AI-ECG performed best at the pulmonary valve position (p=0·02), with an AUROC of 0·85 (95% CI 0·81–0·89), sensitivity of 84·8% (76·2–91·3), and specificity of 69·5% (66·4–72·6). Diagnostic odds ratios did not differ by age, sex, or non-White ethnicity. Taking the optimal combination of two positions (pulmonary and handheld positions), the rule-based approach resulted in an AUROC of 0·85 (0·81–0·89), sensitivity of 82·7% (72·7–90·2), and specificity of 79·9% (77·0–82·6). Using AI-ECG outputs from these two positions, a weighted logistic regression with l2 regularisation resulted in an AUROC of 0·91 (0·88–0·95), sensitivity of 91·9% (78·1–98·3), and specificity of 80·2% (75·5–84·3).

**Interpretation:**

A deep learning system applied to single-lead ECGs acquired during a routine examination with an ECG-enabled stethoscope can detect LVEF of 40% or lower. These findings highlight the potential for inexpensive, non-invasive, workflow-adapted, point-of-care screening, for earlier diagnosis and prognostically beneficial treatment.

**Funding:**

NHS Accelerated Access Collaborative, NHSX, and the National Institute for Health Research.

## Introduction

The escalating worldwide burden of heart failure is compounded by late diagnosis, which both worsens patients' prognoses and increases costs for health systems, primarily through avoidable hospital admissions.^1^, ^2^, ^3^ In the UK, the National Health Service (NHS) Long Term Plan emphasises this shortcoming in care, highlighting that “80% of heart failure is currently diagnosed in hospital, despite 40% of patients having symptoms that should have triggered an earlier assessment”.^4^ Among these patients, about 50% have heart failure with reduced ejection fraction, designated by an echocardiogram-derived left ventricular ejection fraction (LVEF) of 40% or lower.^5^ The prognosis for this patient group continues to improve with advancements in cost-effective drug and device therapies, where timely commencement maximises benefits.^6^, ^7^, ^8^ There is, therefore, an important unmet need for inexpensive and practical point-of-care screening for an LVEF of 40% or lower.

**Research in context**

**Evidence before this study**

Previous studies have applied deep learning methods to highlight the 12-lead ECG as an accurate digital biomarker for changes in left ventricular ejection fraction (LVEF). We searched the Embase and MEDLINE databases for relevant full-text articles written in English published between database inception and July 2, 2021. Our search strings included “ECG” or “EKG” OR “electrocardiogram”, AND “deep learning” OR “neural network” OR “machine learning” AND “prediction” OR “screening” AND “heart failure” or “systolic dysfunction” OR “ejection fraction.” 1284 abstracts were reviewed for suitability and 26 full-text articles retrieved accordingly. We identified ten original research studies that applied deep learning to predict LVEF using 12-lead ECGs as inputs. Six of these studies relate to the Mayo Clinic's algorithm for detecting low LVEF from 12-lead ECG (known as artifical intelligence [AI]-ECG), which has been retrospectively externally validated among Mayo Clinic cohorts and in a Russian population. Most recently, AI-ECG was evaluated in a pragmatic randomised trial that used AI-ECG within Mayo Clinic primary care practices, highlighting an increased rate of LVEF ≤50% diagnosis in the intervention group. The patient profile in all these studies is at least 90% White. There are no studies describing the use of AI-ECG using single-lead ECG input alone for prediction of LVEF of 40% or lower, and specifically no studies using an ECG-enabled stethoscope to instantly derive this input for screening at the point of care.

**Added value of this study**

We have completed the first, to our knowledge, prospective, multicentre study of AI-ECG retrained to use single-lead ECG as input. Furthermore, these inputs were recorded during an ECG-enabled stethoscope examination at universal anatomical landmarks for cardiac auscultation, plus one handheld position. Our participants were UK National Health Service patients attending for transthoracic echocardiography, where the transthoracic echocardiogram-derived LVEF was used as the gold-standard ground truth to test the performance of single-lead AI-ECG. We focused on detection of reduced LVEF (≤40%), given the availability of prognostically beneficial therapies in this group. Our study shows the accuracy of AI-ECG using single-lead inputs and that a 15 s ECG-enabled stethoscope examination can reliably (>93%) record adequate inputs for such analysis. We highlight a position over the pulmonary valve as having the highest area under the curve, and indicate this quality can be further improved by using recordings from two positions combined in either a simple rule-based approach or by using further processing AI-ECG outputs with logistic regression. Importantly, our population has unprecedented racial diversity (41% non-White).

**Implications of all the available evidence**

Our study suggests AI-ECG can be applied for point-of-care detection of reduced LVEF using single-lead ECG alone. Acquiring inputs from an ECG-enabled stethoscope uses a familiar clinical tool with universal workflow, potentially facilitating ease of clinical adoption. Whether the performance of AI-ECG holds true in an unselected screening population will require further prospective studies. The opportunity to screen for reduced LVEF in primary care settings might help to address the reality that reduced LVEF is predominantly diagnosed through hospital admission.

Through the application of artificial intelligence (AI), the 12-lead ECG has been described as an accurate digital biomarker for the stages of LVEF compromise. Previous research by the Mayo Clinic showed that a convolutional neural network (known as AI-ECG), trained on 12-lead ECGs labelled with corresponding echocardiogram-derived LVEF, could detect LVEF of 35% or lower with 86·3% sensitivity and 85·7% specificity.^9^ This AI-ECG model has since been externally validated with 12-lead ECGs in further midwestern (US) cohorts^10^, ^11^, ^12^, and in a Russian population (sensitivity 80·8% and specificity 67·3%).^13^ Most recently, a cluster randomised controlled trial made AI-ECG accessible for 12-lead ECG interpretation in a cohort of Mayo Clinic primary care practices, highlighting an increase in the diagnosis of LVEF of 50% or lower (odds ratio [OR] 1·32 (1·01–1·61).^14^

The emergence of ECG-enabled stethoscopes, capable of recording single-lead ECGs during contact for routine auscultation, highlights an opportunity to apply AI-ECG for point-of-care screening. Beyond accuracy of the algorithm when using single-lead ECG alone, this approach is contingent on these inputs being easy to record and being consistently of adequate quality for attempting AI-ECG interpretation.

We aimed to investigate whether AI-ECG, retrained to use single-lead ECG as input, could interpret recordings from an ECG-enabled stethoscope at anatomical sites established within routine clinical examination, and whether it could detect LVEF of 40% or lower in a previously untested population. Our study tested a hypothesis that LVEF of 40% or lower could be detected at or above the clinically meaningful accuracy of previous 12-lead ECG studies (sensitivity >81% and specificity >67%),^13^ demonstrating that a universal cornerstone of patient encounters—the stethoscope examination—could provide a point-of-care screening opportunity.

## Methods

### Study design and participants

In this prospective, multicentre study, patients were recruited from seven NHS sites (including hospitals and community health centres) that perform transthoracic echocardiography in London, UK. Partients were recruited by 15 operators (six clinicians, six sonographers, and three senior medical students), all of whom received the same training. All adults (aged ≥18 years) attending for transthoracic echocardiogram were eligible to participate (inpatients and outpatients). Patients were attending for transthoracic echocardiogram as part of their routine clinical care, having been referred by clinicians for various standard transthoracic echocardiogram indications, such as investigation of symptoms (eg, breathlessness, peripheral oedema, fatigue, and chest pain) and screening (eg, due to hypertension, arrhythmia, stroke, or suspected valve disease). Patients were not excluded on the basis of the reason for transthoracic echocardiogram referral or patient clinical characteristics.

Written informed consent was obtained from all participants before enrolment. This study was approved by the UK Health Research Authority (reference 21/LO/0051). The protocol is available online.

### AI-ECG algorithm

The model design for 12-lead AI-ECG has been previously described.^9^ Briefly, the model uses a convolutional neural network, trained on 35 970 independent pairings of 12-lead ECG and echocardiograms from the proprietary Mayo Clinic digital data vault. We tested the Mayo Clinic's single-lead version of AI-ECG, which uses similar model architecture retrained with each single-lead ECG extracted from the original 12-lead dataset.

**Figure 1:**
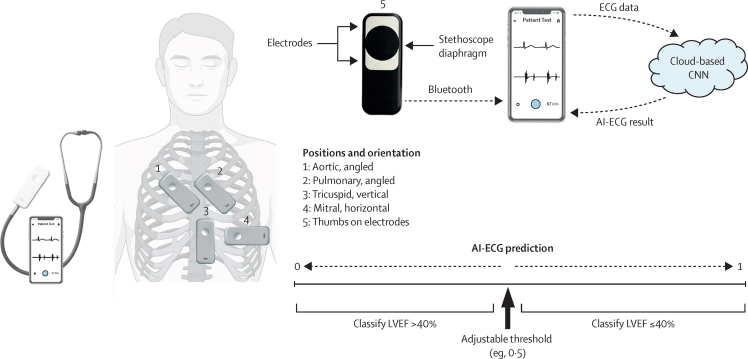
Schematic of ECG-enabled stethoscope and AI-ECG Illustration of anatomical positions for auscultation and position-specific angulation (vector) of ECG-enabled stethoscope; and flow diagram of raw ECG data to cloud-based CNN for interpretation of AI-ECG, with illustration of how raw outputs are classified according to adjustable (optimised) threshold. Anatomical images adapted from BioRender. AI=artificial intelligence. CNN=convolutional neural network. LVEF=left ventricular ejection fraction.

For each single-lead ECG input, the output for the convolutional neural network is a continuous value between 0 and 1, indicating the probability of the condition of interest being present—ie, LVEF of the specified cutoff consistent with heart failure with a reduced ejection fraction (LVEF ≤40%, where prognostically beneficial therapies are available). The AI-ECG threshold can be adjusted along this 0–1 scale (figure 1) according to the LVEF cutoff of interest and trade-off in performance (eg, sensitivity *vs* specificity). The ECG-enabled stethoscope records 15 s of single-lead ECG, where only the first 10 s are analysed by the AI-ECG model. On the basis of a separate deep learning classifier trained on signal quality annotations, which was validated based on ground truth determined by a plurality vote of three cardiologists, ECG recordings are categorised as adequate or inadequate quality to attempt AI-ECG interpretation. AI-ECG will interpret any adequate quality ECG waveform and produce a prediction, regardless of the position or orientation from which the single-lead ECG is being recorded.

### Procedures

Patient recruitment started on Feb 6, 2021. All participants had 15 s of supine, single-lead ECG recorded via two electrodes on a widely available, ECG-enabled stethoscope (Eko DUO; Eko Health, Oakland, CA, USA). All recordings were done within 24 h of transthoracic echocardiogram; almost all were recorded during the same clinical encounter. Members of the research team were unaware of participants' LVEF at the time of recording and remained blinded to these results for the duration of the study.

The ECG-enabled stethoscope has two electrodes on the patient-facing side of the device. Placement on a patient's chest (or handheld) creates a vector for recording ECGs. For simplicity and following a familiar clinical workflow, positions were recorded in sequence at standard anatomical landmarks for auscultation of the aortic, pulmonary, tricuspid, and mitral valves, and at one handheld position. The single, fixed angulation specified for each position was reached via clinical consensus of what was most intuitive and captured various vectors across the five positions. Aortic and pulmonary positions were recorded holding the device angled to the left, with the tricuspid position in a vertical and mitral position in a horizontal orientation (figure 1). Although precordial placement is not identical to electrode positioning for 12-lead ECG, the vectors explored were similar. For example, the pulmonary valve position most closely resembles lead 2 of a 12-lead ECG. Heart sounds (phonocardiograms) were automatically recorded at the same time, but did not serve as inputs for AI-ECG. For the handheld position, patients were asked to place their thumbs on the two electrodes, with the left thumb on the exploring electrode, such that this represented lead 1 of a standard 12-lead ECG.

The ECG-enabled stethoscope transmitted single-lead ECG recordings via Bluetooth for visualisation via an Android or iOS smartphone app (Eko Digital Stethoscope + ECG; Eko Health). The app notified the operator when the ECG signal was of adequate or inadequate quality for attempting interpretation by the algorithm. Only one recording attempt was allowed for each position. The ECG waveform data were analysed in real time by AI-ECG via a cloud-based convolutional neural network, hosted by the device manufacturer using protocols compliant with the Health Insurance Portability and Accountability Act and General Data Protection Regulation. No information was stored on individual users' smartphones. Overall, the full examination took approximately 2 min per patient. Raw AI-ECG predictions for each single-lead ECG were retrieved from the stethoscope manufacturer's online dashboard and combined with a secure, de-identified database containing relevant demographic and clinical variables for each participant. Patients' ethnicity was self-reported from a list of 18 options drawn from the UK Office of National Statistics Census for England.^15^

### Outcomes

Our primary outcome was the identification of patients with an LVEF of 40% or lower from single-lead ECG recordings obtained by the ECG-enabled stethoscope. For diagnostic accuracy assessment, the gold standard was percentage LVEF as measured on a 2D transthoracic echocardiogram acquired by echocardiographers accredited by the British Society of Echocardiography.^16^ LVEF was recorded in line with the same approach taken by the Mayo Clinic for labelling the ground-truth training dataset for AI-ECG. Namely, the first LVEF available from a standard hierarchical sequence: a biplane approach using the Simpson method, a 2D method, or M-mode and, in the absence of any of the preceding, the reported visually estimated LVEF. Where LVEF was reported as a range, the midpoint value was used. LVEF was further binarised according to the LVEF cutoff of interest.

### Statistical analysis

Demographical and clinical variables were summarised for the overall cohort using means and standard deviations. We compared groups stratified by LVEF (>40 *vs* ≤40%) using Student's *t* tests for continuous variables or Pearson's χ^2^ test for categorical variables, as appropriate, with p<0·05 considered statistically significant. The 18 possible options for ethnicity were grouped into White, Black, Asian, mixed, and other.

Using outputs from the AI-ECG model in the interval range of 0–1, performance at classifying LVEF (>40% *vs* ≤40%) was measured for each position by calculating the area under the receiver operating characteristic curve (AUROC), using a reference standard of transthoracic echocardiogram-derived percentage LVEF. We tested the AUROC results between the best and second best performing single position using the DeLong test for significance.^17^ For each position, we also report sensitivity, specificity, negative and positive predictive value, and F1 score at (1) the optimal threshold maximising the sum of sensitivity and specificity (ie, Youden's index), and (2) a restricted threshold that would maximise the sum of sensitivity and specificity, with a minimum sensitivity of 81% and (where possible) a minimum specificity of 67%. 95% CIs are reported using the latter restriction. Using the single-best performing position and compared with the overall population, we also report diagnostic ORs stratified by sex for two age bands (18–69 years and ≥70 years) and by non-White ethnicity. Diagnostic OR is the ratio of positive likelihood ratio (sensitivity / [1 – specificity]) to the negative likelihood ratio ([1 – sensitivity] / specificity). We applied the Breslow-Day test for homogeneity to test for significant (p<0·05) variation in performance. Performance is reported using only ECG recordings of adequate quality to attempt AI-ECG analysis.

Expanding beyond the single-best position alone, performance is also reported when considering the best combination of two positions when using a rule-based approach, where either position predicting LVEF of 40% or lower was a positive test result. Using the dataset of 0–1 values for AI-ECG model predictions from each of the two optimally combined positions as inputs, several classification models (including logistic regression) were tested for predicting LVEF of 40% or lower. These models used 60% of the dataset for training and 40% for testing, and consisted of equal proportions of patients with each LVEF status (>40% *vs* ≤40%; randomly allocated). The best model was selected using five-fold cross validation.

Using predictions from the AI-ECG neural network in the interval range 0–1 for each single-lead ECG, receiver operating characteristic curves were plotted to display performance across a full range of thresholds. We generated a receiver operating characteristic curve summarising the single-best position, rule-based optimal combination of two positions, and best overall classification model. Confusion matrices are presented using the restricted threshold.

All analyses were done in R (version 3.6.1) and Python (version 3.7.6). We used STARD reporting guidelines (checklist included in the appendix pp 6–7). This study is registered with ClinicalTrials.org NCT04601415.

### Role of the funding source

The funders had no role in the study design, data collection, data analysis, data interpretation, or writing of the report.

## Results

**Figure 2:**
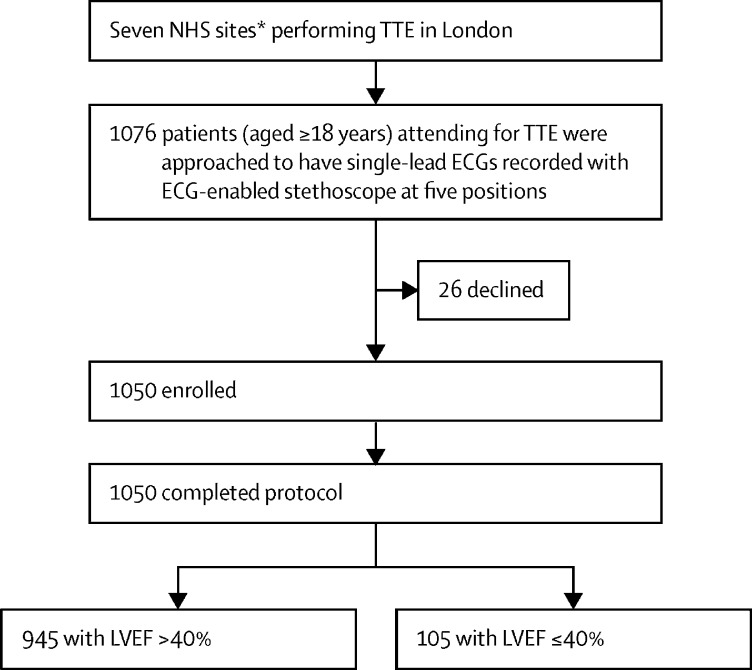
Study profile TTE=transthoracic echocardiogram. LVEF=left ventricular ejection fraction. NHS=National Health Service. *Three hospitals and four community centres.

Between Feb 6 and May 27, 2021, 1050 patients were recruited, of whom 105 (10%) had an LVEF of 40% or lower and 945 (90%) had an LVEF of at least 40% (figure 2). Overall, the mean age was 62 years (17·4); 535 (51%) patients were male and 432 (41%) were non-White. Full ethnicity breakdown is available in the appendix (p 2). Compared with the normal LVEF group (LVEF >40%), the reduced LVEF group (LVEF ≤40%) was older (mean age 62 years [SD 17·5] *vs* 67 years [15·3]) and had fewer female participants (36 [34%] of 105 *vs* 479 [51%] of 945; table 1). Most comorbidities were more prevalent among the reduced LVEF group.

**Table 1:** Baseline characteristics of study participants

		**All participants (n=1050)**	**LVEF >40 group (n=945)**	**LVEF ≤40 group (n=105)**	**p value**
Age, years					
	18–69	636 (61%)	583 (62%)	53 (50%)	0·034
	≥70	414 (39%)	362 (38%)	52 (50%)	..
	Mean (SD)	62 (17·4)	62 (17·5)	67 (15·3)	0·0014
Sex					
	Male	535 (51%)	466 (49%)	69 (66%)	0·0015
	Female	..	..	..	..
Mean TTE LVEF (SD), %		54% (10·3)	57% (5·8)	30% (8·2)	<0·0001
Ethnicity		..	..	..	0·4
	Asian	199 (19%)	176 (19%)	23 (22%)	..
	Black	95 (9%)	84 (9%)	11 (10%)	..
	Mixed	22 (2%)	18 (12%)	<5	..
	Other	116 (11%)	102 (11%)	14 (13%)	..
	White	618 (59%)	565 (60%)	53 (50%)	..
Medical history					
	Hypertension	395 (38%)	338 (36%)	57 (54%)	<0·0001
	Myocardial infarction	102 (10%)	62 (6%)	40 (38%)	<0·0001
	Atrial fibrillation	173 (16%)	146 (15%)	27 (26%)	0·011
	Pacemaker	59 (6%)	43 (5%)	16 (15%)	<0·0001
	Diabetes	224 (21%)	181 (19%)	43 (41%)	<0·0001
	Stroke or transient ischaemic attack	100 (10%)	85 (9%)	15 (14%)	0·11
	Chronic kidney disease	98 (9%)	74 (8%)	24 (23%)	<0·001
	Smoking	148 (14%)	132 (14%)	16 (15%)	0·78
	Excessive alcohol intake	26 (2%)	25 (2·6%)	<5	0·48
	Hypercholesterolaemia	188 (18%)	159 (17%)	29 (28%)	0·0098
	Pregnancy (current)	21 (2%)	21 (2%)	0	0·24
	Chronic obstructive pulmonary disease	57 (5%)	48 (5%)	9 (89%)	0·20

Data are n (%) unless otherwise stated. Characteristics reported in fewer than five participants are shown as <5. p values were calculated via Student's *t* test or Pearson's χ^2^ test. Ethnicity was self-reported from a list of 18 options drawn from the UK Office of National Statistics Census for England.^15^ Full ethnicity breakdown is available in the appendix (p 2). TTE LVEF=transthoracic echocardiogram-derived left ventricular ejection fraction.

Single-lead ECG recordings were attempted at all precordial positions in 1045 (99·5%) of 1050 participants. For the handheld position, this was 1006 (95·8%); reasons for not attempting ECG recording included patients being unable to hold the device (eg, due to previous stroke). Recording of a 15 s ECG of adequate signal quality for attempting AI-ECG interpretation varied across positions, with the aortic (846 [80·6%] of 1050) and pulmonary (979 [93·2%]) positions performing worst and best, respectively (table 2). Taking position 2 as an example, baseline characteristics for age and sex did not differ between those who did and did not have adequate quality recordings (p>0·05).

**Table 2:** Performance characteristics of AI-ECG, by position

	**Adequate ECG, n/N (%)**	**AUC**	**Maximising Se and Sp equally (Youden index)**						**Maximising Se and Sp with rule Se >81, Sp >67, Se >81, or maximising Sp**					
			Threshold	Se	Sp	PPV	NPV	F1 score	Threshold	Se	Sp	PPV	NPV	F1 score
1	846/1050 (80·6%)	0·75	0·370	77·1	60·7	17·3	95·9	0·282	0·345	81·9	53·3	15·8	96·2	0·264
2	979/1050 (93·2%)	0·85	0 ·443	71·7	86·5	37·0	96·3	0·486	0·341	84·8	69·5	23·6	97·4	0·369
3	946/1050 (90·1%)	0·78	0·489	68·1	77·4	24·7	95·5	0·361	0·280	81·9	55·2	16·6	96·3	0·275
4	968/1050 (92·2%)	0·78	0·420	62·9	80·6	26·2	95·0	0·368	0·312	81·4	58·4	17·7	96·4	0·290
5	916/1050 (87·2%)	0·79	0·427	62·8	83·4	27·7	95·5	0·383	0·304	81·4	60·1	17·5	96·8	0·287
2 and 5	864/1050 (82·3%)*	0·85	0·450	82·7	79·9	29·9	87·8	0·439	0·450	82·7	79·9	29·9	87·8	0·439
2 and 5, LR	346/864 (40%)†	0·91	0·497	91·9	80·2	35·1	98·4	0·503	0·497	91·9	80·2	35·1	98·4	0·503

AUC=area under the curve. 1=aortic. 2=pulmonary. 3=tricuspid. 4=mitral. 5=handheld. AI=artificial intelligence. LR=logistic regression. Se=sensitivity. Sp=specificity. PPV=positive predictive value. NPV=negative predictive value.

* Number of patients who had adequate recordings at both position 2 and 5, where a positive AI-ECG result as per threshold was considered a positive test.

† Representing 40% testing dataset from the original 864 participants with both position 2 and 5 recordings.

**Figure 3:**
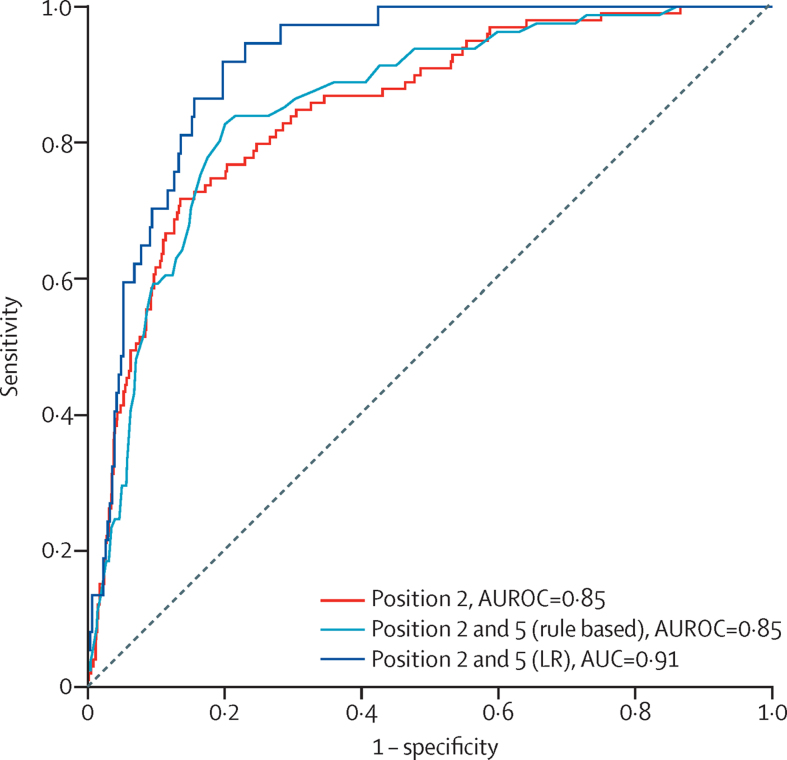
Receiver operating characteristic curves detection of reduced LVEF Data are shown for the single-best performing position (pulmonary), rule-based optimal combination of two positions (pulmonary and handheld), and exploratory logistic regression model with l2 regularisation using AI-enabled ECG outputs from optimal combination of two positions. AUROC=area under the receiver operating characteristic curve. LR=logistic regression. LVEF=left ventricular ejection fraction.

The performance of the AI-ECG algorithm is summarised in table 2. Confusion matrices are presented in table 3. The single-best performing position was over the pulmonary valve, with an AUROC of 0·85 (95% CI 0·81–0·89), sensitivity of 84·8% (76·2–91·3), and specificity of 69·5% (66·4–72·6; figure 3). The second-best position was handheld, with an AUROC of 0·79 (0·74–0·84; p=0·02). When considering the restricted threshold for recordings over the pulmonary valve, the number of false positive results was higher in the LVEF 41–50% range (47 [43%] of 109) than in those with a normal LVEF of 50–70% (215 [26·2%] of 820, p=0·01; appendix p 3). The appendix (p 4) shows differences in model performance among the three operators who recruited the most patients.

**Table 3:** Confusion matrices

	**All participants**	**LVEF ≤40%**	**LVEF >40%**
**Position 2**			
Number	979	99	880
AI-ECG positive	352	84	268
AI-ECG negative	627	15	612
**Positions 2 and 5 (rule based)**			
Number	864	81	783
AI-ECG positive	224	67	157
AI-ECG negative	640	14	626
**Positions 2 and 5 (logistic regression)**			
Number	346	n=37	309
AI-ECG positive	95	34	61
AI-ECG negative	251	3	248

Confusion matrices are displayed according to the restricted threshold for maximising sensitivity and specificity, with rule sensitivity >81, specificity >67; or sensitivity >81, maximising specificity. AI=artificial intelligence. LVEF=left ventricular ejection fraction.

The pulmonary and handheld positions performed best when combined using a rule-based approach: either one or both predicting LVEF of 40% or lower being considered a positive test. For this analysis, 864 (82·3%) of 1050 patients had adequate quality single-lead ECG for attempted AI-ECG prediction at both positions. The resultant AUROC was 0·85 (95% CI 0·81–0·89), with 82·7% (72·7–90·2) sensitivity and 79·9% (77·0-82·6) specificity.

The model with the best performance used weighted logistic regression with l2 regularisation. We used data from 864 patients (number of patients with adequate ECG recordings at both pulmonary and handheld positions): 518 (60%) for training and 346 (40%) for testing. Using AI-ECG outputs from these two positions, a weighted logistic regression with l2 regularisation resulted in an AUROC of 0·91 (0·88–0·95), sensitivity of 91·9% (78·1–98·3), and specificity of 80·2% (75·5–84·3).

**Figure 4:**
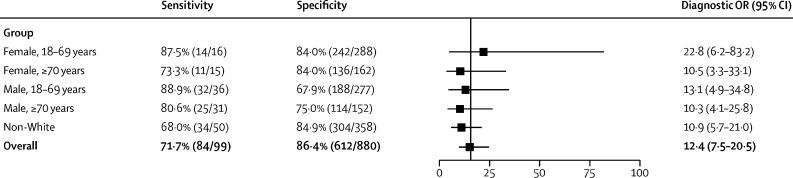
Convolutional neural network's sensitivity and specificity to detect LVEF ≤40% Data are tabulated across a range stratified into age bands by sex, and by non-White ethnicity, using results from the pulmonary position at the threshold maximising the sum of sensitivity and specificity. The diagnostic OR and associated 95% CI is shown for each group and for the overall study sample. For sensitivity, data presented in brackets represents the number of patients in each group who had a positive result, with the denominator the number of patients with LVEF ≤40%. For specificity, this is the number of patients with a negative result, with the denominator patients with LVEF>40%. OR=odds ratio. LVEF=left ventricular ejection fraction.

The performance of the AI-ECG algorithm stratified by sex and age (18–69 years *vs* ≥70 years), and by non-White ethnicity, is presented in figure 4. Compared with the overall diagnostic OR for the whole study population, no significant differences were seen.

## Discussion

This observational, prospective, multicentre study showed for the first time the performance of AI-ECG for detecting LVEF of 40% or lower using only single-lead ECGs recorded during an ECG-enabled stethoscope examination. Our study of 1050 patients undergoing transthoracic echocardiogram found that a single-best position, two best positions combined, and an exploratory logistic regression model attained AUROCs of 0·85, 0·85, and 0·91, respectively. These results suggest that the stethoscope examination, a universal component of the clinician–patient interaction, can be used as a screening tool for LVEF of 40% or lower by combining ECG recording and AI at the point of care.

From a public health perspective, combining AI with an ECG-enabled stethoscope examination for low-cost screening for LVEF of 40% or lower fulfils key criteria for a screening programme, including the underlying condition being a public health priority,^18^ involving a latent or early symptomatic phase,^19^ and for which evidence-based therapies are available. Further evaluation of the potential cost-effectiveness and effects on patient outcomes will be needed, especially in conjunction with established screening tests for heart failure, such as natriuretic peptide blood tests, which could further improve predictive capability. Easily available clinical tabular variables, such as age, sex, blood pressure, or the presence of comorbid illness, might further improve the model output and aid in the identification of systolic dysfunction. In the clinic, pre-test probability is likely to be greatest among those with heart failure with reduced ejection fraction symptoms (eg, breathlessness, ankle oedema, and fatigue). However, these are non-specific symptoms and can result in a host of other acute or chronic conditions being investigated first. Here, where a stethoscope examination would always be indicated, delays to diagnosis might be avoided by flagging the possibility of heart failure with a reduced ejection fraction early. Given the substantial expense of echocardiography and the NHS-wide shortage of echocardiographers,^20^ the high negative predictive value (97%) could also enable resource prioritisation.

Successful system-wide adoption of any AI tool will require trust from patients and clinicians, and behavioural change in the latter to both adopt and follow recommendations from algorithms.^21^, ^22^ The unknown, black box nature of the neural network means that the specific ECG features that determine individuals' classification of LVEF status are not obvious, although it probably draws on established pathological effects of reduction in LVEF on the ECG.^23^, ^24^, ^25^ Similar to the application of neural networks for predicting coronary artery disease from retinal imaging,^26^ our study uses AI for the interpretation of a digital biomarker beyond the capacity of human skill. We include examples of single-lead ECGs at the pulmonary position classified by the optimised threshold as true positive, false positive, and false negative, for visual inspection in the appendix (p 5). Consistent features and weighting are not immediately obvious. Reassuringly, in our study population, performance of the algorithm did not differ by age, sex, or non-White ethnicity.

An external validation study of AI-ECG using 12-lead inputs reported an AUROC of 0·82^13^, compared with 0·93 for the original internal validation study.^9^ Our findings using only single-lead ECG inputs, therefore, compare favourably. The low positive predictive value should be interpreted in the context of both the selected study population—patients attending for ECG and, therefore, more likely to have abnormal ECG features that risk a false positive result—and that false positives occurred most frequently in the LVEF 41–50% range. This is within the diagnostic spectrum of heart failure where, from a clinical perspective, further investigation would be warranted, entailing minimal-risk natriuretic peptide blood testing. Therefore, a key challenge will be how to select and define specific thresholds and cutoff points. For example, more specific cutoffs could optimise against false positive rates, but at the expense of lower sensitivity. Population-specific cutoffs might be necessary to optimise test performance for differing demographical and disease profiles, including different underlying disease prevalence.

Defining the unit of examination (one or multiple positions) will also be important. We have shown that performance was moderately improved when applying AI-ECG at two positions. Considering the four universal positions for stethoscope examination (plus one handheld) for each patient, we obtained five raw AI-ECG outputs. When exploring just two of these, inputting AI-ECG values into a logistic regression model showed substantial improvement in performance. Notably, such an approach should not compromise the tool's ease of use by requiring an end user to take multiple recordings or manually input data into a further model, which should be avoidable through automation and considered user experience design. Displaying percentage LVEF or raw predictions as a continuous variable might empower individual clinician choice on the appropriate threshold for ordering further investigations on a case-by-case basis; however, as a decision aid for non-specialists, this feature might be less desirable.

The original internal validation study^9^ (12-lead ECG) identified a four-times increase of developing an LVEF of 35% or lower in subsequent years if AI-ECG predicted an ejection fraction of 35% or lower, but echocardiogram-derived LVEF was at least 35%. This finding highlights the possibility that ECG changes might predate deterioration in LVEF detectable by echocardiography. Accordingly, we will perform long-term follow-up of clinical outcomes for false positives in our study cohort to investigate if AI-ECG can propose a cohort for surveillance.

Given substantial concerns and criticisms of validation studies of health-related AI tools,^27^, ^28^, ^29^ our study design has several strengths. First, data were collected prospectively across multiple real-world settings and by many operators. Second, the authors are independent of the groups who developed both AI-ECG and the ECG-enabled stethoscope. Third, our study population was unrelated to the training cohort, and our sample's ethnic diversity (41% non-White) is unmatched by previous, retrospective external validation studies of AI-ECG (which were <10% non-White). Fourth, beyond testing the performance of AI-ECG alone, we evaluated a form factor and workflow for front-line clinical delivery that has several advantages over 12-lead ECGs. Namely, the recording of ECGs during auscultation over the single-best (pulmonary) position achieved adequate recordings to attempt prediction in at least 93% of patients (*vs* 87% for handheld position), taking 15 s to complete and requiring minimal training. Fifth, use of AI and an ECG-enabled stethoscope upgrades a familiar tool already in daily clinical use. This potentially overcomes barriers, such as maintained use by clinicians, previously identified as a challenge for other devices capable of recording single-lead ECGs.^30^ Such a tool could be particularly impactful in the busy primary care setting, given that, among the 80% of patients diagnosed with heart failure in hospital, 40% have had a recent primary care encounter with symptoms of heart failure that would have warranted a stethoscope examination.^1^ This approach would also be of value in low-income countries with health systems where access to cardiological care and imaging is scarce.^21^, ^31^ Finally, beyond the scope of this study, the dual acquisition of precordial ECG and phonocardiogram (heart sounds) highlights an opportunity to also screen for further priority cardiovascular diseases, such as valvular heart pathology, using AI-enabled phonocardiography.^32^ Similarly, the 15 s single-lead ECG offers an opportunity for the detection of atrial fibrillation, either by visual inspection, or also supported by AI.^33^, ^34^ Improvements in accuracy for predicting reduced LVEF might be achievable by combined AI analysis of synchronous ECG and phonocardiogram waveforms.

Our study has limitations. First, the patient cohort is not fully representative of a screening population, where lower prevalence of an LVEF of 40% or lower could influence performance characteristics, particularly positive predictive values. Although the disease profile of our population probably reflects those who would benefit most from screening for reduced LVEF, further studies are needed, particularly in primary care settings. Further investigation would also address the current paucity of data describing prevalence of asymptomatic disease. Second, without comprehensive access to all participants' electronic health records to determine any previously normal LVEF, we are unable to precisely characterise how many participants were flagged as positive by AI-ECG as part of an index diagnosis of heart failure with a reduced ejection fraction. Third, there is established inter-operator variability in measurement of LVEF from echocardiography, giving rise to the possibility that some participants close to the LVEF 40% borderline were misclassified. Further studies with a higher number of operators across the wider clinical workforce will be required to determine if the device is universally easy to use. Lastly, we have not tested for reproducibility. There will have been inter-operator variability in the precise position and angulation of the ECG-enabled stethoscope, although this variability reflects the reality of clinical practice.

In summary, our study found that AI-ECG could identify patients with reduced LVEF (≤40%) from single-lead ECG inputs. Through use of an ECG-enabled stethoscope, we highlight an AI algorithm embedded in a familiar clinical tool that fits into routine and universal clinical workflows. Given the frequent clinical encounters of undiagnosed patients before index hospital admission for heart failure, the stethoscope examination has the potential to be a point-of-care screening opportunity, and through further AI algorithms, to become a tool for comprehensive detection of cardiovascular disease.

## Data sharing

Data collected during our study can be made available as part of further research collaborations. Interested parties should contact the corresponding author (NSP). Any data sharing will be subject to meeting data protection rules and regulations of Imperial College London and Imperial College Healthcare NHS Trust. Code used for the analysis of this study is freely available on GitHub. The code that constitutes the AI-ECG algorithm is proprietary to the Mayo Clinic, which has licensed AI-ECG to Eko Health for use with the ECG-enabled stethoscopes. The authors of this work did not have access to the code for the AI-ECG model and associated Mayo Clinic data.

## Declaration of interests

We declare no competing interests.
