# Supplementary material for: Point-of-care screening for heart failure with reduced ejection fraction using artificial intelligence during ECG-enabled stethoscope examination in London, UK: a prospective, observational, multicentre study
Source: Lancet Digit Health. 2022 Jan 5;4(2):e117–25. doi: 10.1016/S2589-7500(21)00256-9 (PMC8789562; doi:10.1016/S2589-7500(21)00256-9)
Supplement: Supplementary appendix [file mmc1.pdf]

THE LANCET
Digital Health

Supplementary appendix

This appendix formed part of the original submission and has been peer reviewed. We post it as supplied by the authors.

Supplement to: Bachtiger P, Petri CF, Scott FE, et al. Point-of-care screening for heart failure with reduced ejection fraction using artificial intelligence during ECG-enabled stethoscope examination in London, UK: a prospective, observational, multicentre study. *Lancet Digit Health* 2022; published online Jan 5. https://doi.org/10.1016/S2589-7500(21)00256-9.

Supplementary Appendix

Bachtiger et al. 2021. Point-of-Care Screening for Heart Failure with Reduced Ejection Fraction Using Artificial Intelligence During ECG-Enabled Stethoscope Examination: Independent Real-World Prospective Multicentre External Validation Study

Contents:

Supplementary item	Page
Supplementary table 1. *Full breakdown of self-reported race/ethnicity according to ONS Census list for England.*	2
Supplementary table 2. *Comparison of false positive rates in LVEF 41-50% vs. LVEF 50-70% group.*	3
Supplementary Table 3*. Differences in model performance among the three operators who recruited the most patients.*	4
Supplementary figure 1. *Single-lead ECG recordings from position 2 (pulmonary valve, angled).*	5
STARD checklist	6

cc BY SA

Supplementary table 1. *Full breakdown of self-reported race/ethnicity according to ONS Census list for England.*

**Race/ethnicity, total n = 1,050**	**LVEF>40**	**LVEF≤40**	**Overall**
African	31 (3.3%)	3 (2.9%)	34 (3.2%)
Any other Black/ African/Caribbean background	27 (2.8%)	6 (5.7%)	33 (3.1%)
Any other ethnic group	83 (8.7%)	13 (12%)	96 (9.1%)
Any other Mixed/Multiple ethnic background	7 (0.7%)	2 (1.9%)	9 (0.9%)
Any other White background	118 (12%)	12 (11%)	130 (12%)
Arab	20 (2.1%)	1 (1.0%)	21 (2.0%)
Bangladeshi	3 (0.3%)	0 (0%)	3 (0.3%)
Caribbean	26 (2.7%)	2 (1.9%)	467 (44%)
Chinese	9 (0.9%)	1 (1.0%)	28 (2.7%)
English / Welsh / Scottish / N Irish/ British	429 (45%)	38 (36%)	10 (0.9%)
Indian	59 (6.2%)	11 (10%)	70 (6.6%)
Irish	21 (2.2%)	3 (2.9%)	24 (2.3%)
Other Asian background	94 (9.9%)	7 (6.7%)	101 (9.6%)
Pakistani	11 (1.2%)	4 (3.8%)	15 (1.4%)
White and Asian	2 (0.2%)	1 (1.0%)	3 (0.3%)
White and Black African	6 (0.6%)	0 (0%)	6 (0.6%)
White and Black Caribbean	3 (0.3%)	1 (1.0%)	4 (0.4%)

CC BY SA

Supplementary table 2. *Comparison of false positive rates in LVEF 41-50% vs. LVEF 50-70% group.*

**Group**	**LVEF 41-50%**	**LVEF 50-70%**	**Total**
Positive AI-ECG	47 (43.1%)	215 (26.2%)	262
Negative AI-ECG	62 (56.9%)	605 (73.8%)	667
Total	109	820	

Supplementary Table 3. *Differences in model performance among the three operators who recruited the most patients.*

-	***Position 2 (pulmonary***	*-*	***Maximising sensitivity and specificity with rule Se>81, Sp>67; or Se>81, maximising Sp***				
Operator	***Adequate recording***	***AUC (CI)***	***Se***	***Sp***	***PPV***	***NPV***	***F1***
1	*198/218 (90.7%)*	*0.80 (0.70 - 0.88)*	*77.8 (52.3 – 96.6)*	*67.4 (60.0 - 74.2)*	*19.4*	*96.8*	*0.311*
2	*192/197 (97.4%)*	*0.87 (0.77 - 0.94)*	*85.0 (62.1 – 96.7)*	*75.6(68.3 – 82.0)*	*29.8*	*97.6*	*0.442*
3	*182/204 (89.1%)*	*0.88 (0.78 - 0.96)*	*78.6 (48.2 – 9.3)*	*72.9 (65.4 – 79.4)*	*19.6*	*97.6*	*0.314*

cc BY SA

Supplementary figure 1. *Single-lead ECG recordings from position 2 (pulmonary valve, angled). Recordings were categorised as true positive, false positive, true negative, and false negative based on the raw AI-ECG output (between 0 and 1), relative to an optimum classification threshold of 0.341 (achieving minimum sensitivity and specificity of 81% and 67%, respectively). ECGs displayed at recording calibration of 25mm/second, 10mm/mV.*

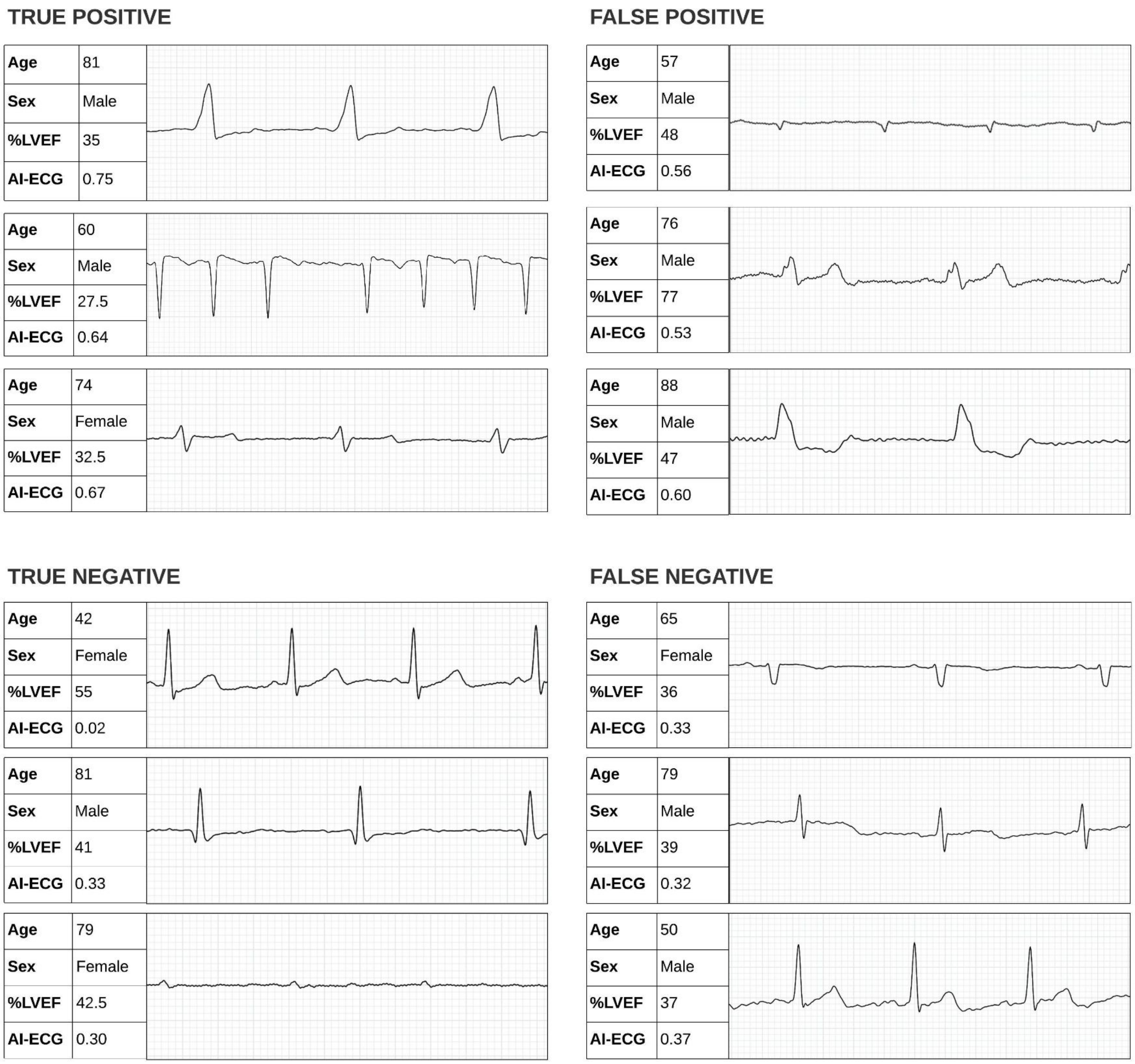

CC BY SA

STARD Checklist

Section & Topic	No	Item	Reported on page #
**TITLE OR ABSTRACT**			
	**1**	Identification as a study of diagnostic accuracy using at least one measure of accuracy (such as sensitivity, specificity, predictive values, or AUC)	2
**ABSTRACT**			
	**2**	Structured summary of study design, methods, results, and conclusions (for specific guidance, see STARD for Abstracts)	2
**INTRODUCTION**			
	**3**	Scientific and clinical background, including the intended use and clinical role of the index test	4
	**4**	Study objectives and hypotheses	4
**METHODS**			
Study design	**5**	Whether data collection was planned before the index test and reference standard were performed (prospective study) or after (retrospective study)	5
Participants	**6**	Eligibility criteria	5
	**7**	On what basis potentially eligible participants were identified (such as symptoms, results from previous tests, inclusion in registry)	5
	**8**	Where and when potentially eligible participants were identified (setting, location and dates)	5
	**9**	Whether participants formed a consecutive, random or convenience series	5
Test methods	**10a**	Index test, in sufficient detail to allow replication	5
	**10b**	Reference standard, in sufficient detail to allow replication	6
	**11**	Rationale for choosing the reference standard (if alternatives exist)	6
	**12a**	Definition of and rationale for test positivity cut-offs or result categories of the index test, distinguishing pre-specified from exploratory	6
	**12b**	Definition of and rationale for test positivity cut-offs or result categories of the reference standard, distinguishing pre-specified from exploratory	6
	**13a**	Whether clinical information and reference standard results were available to the performers/readers of the index test	6
	**13b**	Whether clinical information and index test results were available to the assessors of the reference standard	6
Analysis	**14**	Methods for estimating or comparing measures of diagnostic accuracy	7
	**15**	How indeterminate index test or reference standard results were handled	7
	**16**	How missing data on the index test and reference standard were handled	7
	**17**	Any analyses of variability in diagnostic accuracy, distinguishing pre-specified from exploratory	7
	**18**	Intended sample size and how it was determined	N/A
**RESULTS**			
Participants	**19**	Flow of participants, using a diagram	8
	**20**	Baseline demographic and clinical characteristics of participants	9
	**21a**	Distribution of severity of disease in those with the target condition	9
	**21b**	Distribution of alternative diagnoses in those without the target condition	9
	**22**	Time interval and any clinical interventions between index test and reference standard	N/A

cc BY SA

Test results	**23**	Cross tabulation of the index test results (or their distribution) by the results of the reference standard	10
	**24**	Estimates of diagnostic accuracy and their precision (such as 95% confidence intervals)	10
	**25**	Any adverse events from performing the index test or the reference standard	N/A
**DISCUSSION**			
	**26**	Study limitations, including sources of potential bias, statistical uncertainty, and generalisability	11
	**27**	Implications for practice, including the intended use and clinical role of the index test	12
**OTHER INFORMATION**			
	**28**	Registration number and name of registry	2
	**29**	Where the full study protocol can be accessed	4
	**30**	Sources of funding and other support; role of funders	2

CC BY SA
